# Aldaulactone – An Original Phytotoxic Secondary Metabolite Involved in the Aggressiveness of *Alternaria dauci* on Carrot

**DOI:** 10.3389/fpls.2018.00502

**Published:** 2018-05-03

**Authors:** Julia Courtial, Latifa Hamama, Jean-Jacques Helesbeux, Mickaël Lecomte, Yann Renaux, Esteban Guichard, Linda Voisine, Claire Yovanopoulos, Bruno Hamon, Laurent Ogé, Pascal Richomme, Mathilde Briard, Tristan Boureau, Séverine Gagné, Pascal Poupard, Romain Berruyer

**Affiliations:** ^1^IRHS, INRA, AGROCAMPUS-Ouest, Université d’Angers, SFR 4207 QUASAV, Beaucouzé, France; ^2^Substances d’Origine Naturelle et Analogues Structuraux, SFR4207 QUASAV, UNIV Angers, Université Bretagne Loire, Beaucouzé, France; ^3^PHENOTIC Platform, IRHS, INRA, AGROCAMPUS-Ouest, Université d’Angers, SFR 4207 QUASAV, Beaucouzé, France; ^4^Département de Biologie, Faculté des Sciences, Université d’Angers, Angers, France

**Keywords:** *Alternaria* leaf blight, aggressiveness, fungal pathogenicity, *in vitro* culture, phytotoxin, quantitative disease resistance, 10-membered benzenediol lactone, decalactone

## Abstract

Qualitative plant resistance mechanisms and pathogen virulence have been extensively studied since the formulation of the gene-for-gene hypothesis. The mechanisms involved in the quantitative traits of aggressiveness and plant partial resistance are less well-known. Nevertheless, they are prevalent in most plant-necrotrophic pathogen interactions, including the *Daucus carota*–*Alternaria dauci* interaction. Phytotoxic metabolite production by the pathogen plays a key role in aggressiveness in these interactions. The aim of the present study was to explore the link between *A. dauci* aggressiveness and toxin production. We challenged carrot embryogenic cell cultures from a susceptible genotype (H1) and two partially resistant genotypes (I2 and K3) with exudates from *A. dauci* strains with various aggressiveness levels. Interestingly, *A. dauci*-resistant carrot genotypes were only affected by exudates from the most aggressive strain in our study (ITA002). Our results highlight a positive link between *A. dauci* aggressiveness and the fungal exudate cell toxicity. We hypothesize that the fungal exudate toxicity was linked with the amount of toxic compounds produced by the fungus. Interestingly, organic exudate production by the fungus was correlated with aggressiveness. Hence, we further analyzed the fungal organic extract using HPLC, and correlations between the observed peak intensities and fungal aggressiveness were measured. One observed peak was closely correlated with fungal aggressiveness. We succeeded in purifying this peak and NMR analysis revealed that the purified compound was a novel 10-membered benzenediol lactone, a polyketid that we named ‘aldaulactone’. We used a new automated image analysis method and found that aldaulactone was toxic to *in vitro* cultured plant cells at those concentrations. The effects of both aldaulactone and fungal organic extracts were weaker on I2-resistant carrot cells compared to H1 carrot cells. Taken together, our results suggest that: (i) aldaulactone is a new phytotoxin, (ii) there is a relationship between the amount of aldaulactone produced and fungal aggressiveness, and (iii) carrot resistance to *A. dauci* involves mechanisms of resistance to aldaulactone.

## Introduction

The genetics of plant–pathogen interactions is classically described as involving a combination of qualitative and quantitative factors. On the qualitative level, the compatibility or incompatibility of the interaction is jointly dependent on the plant resistance genes and pathogen virulence genes. When the interaction is compatible, symptoms appear and the pathogen completes its biological cycle. Nevertheless, the intensity of the disease can be highly variable as it depends on a combination of environmental and genetic factors, with the latter concerning the QDR of the host plant and the aggressiveness of the pathogen. Plant resistance and pathogen virulence determinisms have both been extensively studied from a qualitative standpoint, resulting in the development of general models of these mechanisms, such as the zigzag model (Dangl and Jones, 2006) and the invasion model (Cook et al., 2015). On the quantitative level, scant knowledge is available on the genetic determinism of aggressiveness. For different reasons that have already been reviewed with regard to many phyla (Pariaud et al., 2009), it is quite difficult to find aggressiveness quantitative trait loci (QTLs) in fungal pathogens. QTL mapping of aggressiveness has thus only been performed in a very narrow set of pathogens, e.g., *Gibberella zeae* (Cumagun et al., 2004) and *Heterobasidion annosum* sensu lato (Lind et al., 2007). Conversely, the genetic determinism of QDR has been extensively studied among crop plants, resulting in the detection of a huge number of quantitative resistance loci (QRLs) in a many plant-pathogen interactions (St. Clair, 2010).

No comprehensive model of partial resistance has been developed to date. Nevertheless, Poland et al. (2009) proposed six hypothetical resistance mechanisms: (i) morphological variation, (ii) involvement of microbial triggered immunity, (iii) involvement of chemical warfare, (iv) involvement of the signal transduction pathways involved in effector triggered immunity, (v) QRLs as a weak version of resistance genes, and (vi) new mechanisms. Fully characterized or strong candidate genes underlying more than 15 different QRLs have been uncovered since then (**Supplementary Table S1**). Amongst the mechanisms proposed by Poland, some are over-represented in this currently small set. Six occurrences of QDR genes show strong homologies with resistance genes (**Supplementary Table S1**). Similarly, nine occurrences of QDR genes encode proteins with no known function or with predicted structures that were not previously known to be involved in plant pathogen resistance (**Supplementary Table S1**). Oddly enough, other classes are still lacking characterized genes. For example, no QDR genes have been found to be involved in chemical warfare, i.e., exchange of secondary metabolites between the plant and the fungus. This is surprising, since chemical warfare is known to be an important part of plant–pathogen interactions, in particular those involving fungal pathogens (Pusztahelyi et al., 2015). Among secondary metabolites produced by fungal pathogens, toxins are well-known to be crucial determinants of pathogenicity, especially in necrotrophic fungi (Horbach et al., 2011), such as *Alternaria* species (Thomma, 2003; Meena et al., 2017a). Aggressiveness QTLs have been found at least once to be strongly linked with known toxin production genes (Cumagun et al., 2004). Moreover, resistance genes have sometimes been found to encode proteins involved in toxin resistance. Two classical examples of such resistance mechanisms have been reported in *Cochliobolus carbonum*/maize (Johal and Briggs, 1992) and *Alternaria alternata* f. sp. *lycopersici*/tomato (Spassieva et al., 2002) pathosystems. Toxins were also found to be heavily involved in specific partial resistance in the *Corynespora cassiicola*/rubber tree pathosystem (Barthe et al., 2007). More recently, toxin resistance was found to be correlated with QDR in several pathosystems, including the *Stemphylium solani*/garlic pathosystem (Zheng et al., 2010) and, in our laboratory, the *Alternaria dauci*/*Daucus carota* pathosystem (Lecomte et al., 2014).

Although *A. dauci* can cause symptoms in a large range of dicotyledonous plants (Boedo et al., 2012), this fungal species is mainly known as a pathogen responsible for *Alternaria* leaf blight, i.e., the most prevalent carrot foliar disease. There are many strains of this fungus with highly variable aggressiveness levels (Boedo et al., 2012). Known resistant carrot genotypes show classical horizontal resistance, i.e., the resistance is quantitative, with multigenic determinism involving major QTLs (Le Clerc et al., 2015) conferring resistance to a large number of *A. dauci* isolates. Partially resistant carrot cultivar showed qualitative resistance diversity (Boedo et al., 2010). In other resistance characterization studies (Boedo et al., 2008; Lecomte et al., 2011), we explored several hypotheses including the involvement of chemical warfare in both fungal aggressiveness and plant partial resistance. We thus showed that plant secondary metabolites, such as the phytoanticipin falcarindiol or the phytoalexin 6-methoxymellein, could be involved in partial resistance, but perhaps also in host specificity, since *Alternaria* species that are pathogenic in carrot (*A. dauci*, *A. radicina*) were much less affected by 6-methoxymellein than *A. brassicicola* which is pathogenic in Brassicaceae (Lecomte et al., 2012). More recently, we detected a link between toxins and partial resistance, i.e., partial resistance to *A. dauci* was correlated with *in vitro* cultured carrot cell resistance to fungal exudates (Lecomte et al., 2014). Hydrophobic compounds from these exudates were strongly phytotoxic when applied to cells of *A. dauci*-susceptible carrot genotypes (Lecomte et al., 2015).

The aim of the present study was to explore the link between toxin production and *A. dauci* aggressiveness. The toxicity of fungal exudates from several *A. dauci* strains with different levels of aggressiveness was evaluated in *in vitro* cultured carrot cell suspensions. Fungal exudates were extracted with ethyl acetate. UV profiles of these extracts were determined using high performance liquid chromatography-diode array detector (HPLC-DAD) analysis. Correlations between major peak intensities and fungal aggressiveness were measured. From the major component most correlated with fungal aggressiveness, we purified and characterized a new compound, that we named aldaulactone. The toxicity of aldaulactone was then determined at concentrations measured in *A. dauci* exudates.

## Materials and Methods

### Fungal Exudate Production and Extraction

The fungal material used in this study was obtained from four *A. dauci* strains (AUS001, FRA001, FRA017, and ITA002) collected as described in (Boedo et al., 2012) and an *A. brassicicola* strain (Abra 43) collected as described in Iacomi-Vasilescu et al. (2004). All the fungal material presented here is freely available from the COMIC collection (COMIC – IRHS, 42 rue Georges Morel, 49070 Beaucouzé cedex, France) under the numbers COMIC C0002 (FRA001), COMIC C0003 (FRA017), COMIC C0004 (ITA002), COMIC C0005 (AUS001) and COMIC C0006 (Abra 43). *A. brassicicola* is not known to be pathogenic in carrot. Carrots infected with Abra 43 conidia did not show symptoms during preliminary greenhouse experiments (results not shown). Boedo et al. (2012) assessed strain aggressiveness by measuring the percentage of NLA. AUS001 showed weak aggressiveness, FRA001 and FRA017 medium aggressiveness and ITA002 high aggressiveness on carrot. Fungal strains were grown in petri dishes, as described in (Lecomte et al., 2012). To obtain fungal exudates, 100 mL liquid cultures were prepared in 250 mL erlenmeyer flasks. Potato dextrose broth (Atlas, 2010) was used unless otherwise specified. Carrot juice medium (Lecomte et al., 2014) was used in some specified cases. Liquid medium was inoculated with either conidial suspension to reach a final concentration of 5 ⋅ 10^3^ conidia.mL^-1^ or 10 colonized agar plugs of 5 mm dia. ground in an MSE homogenizer (Measuring & Scientific Equipment Ltd., Spenser Street, London SW1E) set at 10,000 rpm for 1 min. These liquid cultures were grown in the dark for 60 h at 24°C on an orbital shaker set at 125 rpm. Raw exudates (REs) were recovered by serial filtrations of fungal culture liquid phases, through Sefar Nitex (Sefar AG, Heiden, Switzerland) nylon membranes of the following decreasing porosities: 200, 50, and 1 μm. They were then dried and stored as previously described in Lecomte et al. (2014). Organic exudates (OEs) were derived from REs by liquid–liquid extraction, dried, evaporated and then stored as described in Lecomte et al. (2014). The dried OE weight (OEW) was then measured using a precision scale. Extracts similarly obtained from mock cultures incubated in the same conditions were called “mock extracts.” The whole experiment was repeated thrice.

### HPLC Analysis of Fungal Exudate OEs

The OEs were dissolved in HPLC grade methanol at 5 mg.mL^-1^, centrifuged for 10 min at 13,500 rpm. Supernatants were transferred into HPLC vials and chromatographic profiles were obtained using an Agilent Technologies HPLC system (Santa Clara, CA, United States) equipped with a quaternary HPLC pump, degasser, autosampler and DAD. The HPLC mobile phase consisted of water (solvent A) and methanol (solvent B). The solvent gradient started with 90% A/10% B at 0 min and rose to 100% B at 25 min. Finally, 100% B was added for 10 more min, and then 100% A for 1 min. The flow rate was 0.7 mL.min^-1^, the injection volume was 10 μL and the eluent was detected at 233, 254, and 285 nm. All HPLC analyses were performed at 25°C on a Uptispher ODB15QK C18 column (3 μm; 150 mm × 4.6 mm). Major peaks were selected as follows: at each wavelength, a threshold corresponding to 20% of absorption observed in the highest peak was defined. Retention times of major peaks were identified for each of the five strains and mock extract, and an area under a curve (AUC) was calculated using Chem Station Open Lab software (Agilent Technologies, Santa Clara, CA, United States) for each extract at each retention time and each wavelength. When peaks at similar retention times were visible at different wavelengths, AUC correlation coefficients were measured. Peaks were considered to correspond to a single candidate compound when the minimum correlation coefficients (*r*) were above 0.9, and thus a mean AUC was computed from each wavelength. When r was below 0.9, AUCs from the two least correlated peaks were considered separately as candidate compounds. Linear regression was then used to compare OEW, and AUC and OEW × AUC for each candidate compound, with previously reported aggressiveness data (Boedo et al., 2012). All statistical analyses were performed using R v 3.2.4 software (R Core Team, 2016).

### Aldaulactone Purification and Characterization

Aldaulactone purification and characterization was performed from cultures of *A. dauci* FRA001 and ITA002 strains. Liquid cultures and OE extractions were performed as previously, except that the scale of the liquid culture ranged from 10 × 100 mL in 250 mL erlenmeyer flasks, to 4 × 1 L in 5 L erlenmeyer flasks. Purification of aldaulactone from the obtained OE was achieved using the flash chromatography technique in an IntelliFlash 310 (Analogix) apparatus with pre-packed silica gel columns (Macherey-Nagel Chromabond^®^ Flash RS column) and a cyclohexane/acetone mixture as mobile phase (9/1 to 6/4 gradient). ^1^H and ^13^C NMR along with 2D NMR data were obtained on a JEOL JNM-ECZS 400 MHz spectrometer (400 and 100 MHz, respectively) in deuterated chloroform with residual CHCl_3_ signal as a reference.

### Quantitative HPLC Method for Determining the Aldaulactone Concentration

Aldaulactone quantification in Abra 43, FRA001 and ITA002 OEs from four 100 mL fungus cultures was performed using external standard calibration in HPLC. OEs were obtained and dissolved as previously described in section “Fungal Exudate Production and Extraction” and purified aldaulactone was dissolved in HPLC grade methanol at 0.102, 0.28, 0.41, and 0.56 mg.mL^-1^. These solutions were injected and analyzed in triplicate using an HPLC-DAD method. Areas of peak at retention time = 19.1 ± 0.06 min were obtained after chromatogram extraction at 305 nm to produce a calibration curve using linear regression (*r*^2^ = 0.949). The aldaulactone concentration in each OE was then calculated from the 19.1 ± 0.06 min peak area using the linear regression equation.

### Plant Material, Embryogenic Cell Cultures and Treatments

The three already described breeding material *D. carota* genotypes were K3, I2 and H1 (Le Clerc et al., 2015). Briefly, H1 plants were obtained by self-pollinating a single plant of an *Alternaria* leaf blight susceptible S3 line. I2 and K3 were obtained in the same fashion from two genetically different partially resistant S2 lines. Seeds were surface disinfected for 5 min with 70% (v/v) ethanol, followed by immersion in a 25% (v/v) commercial bleach solution for 20 min, subsequently washed three times with sterilized twice distilled water, and then placed in 10 cm × 15 cm glass jars containing solidified B5 Gamborg medium (Gamborg et al., 1976) supplemented with 30 g.L^-1^ sucrose, and 3 g.L^-1^ phytagel with the pH adjusted to 5.8 (B5 solid medium). Jars were maintained in a climatic chamber under a 16 h photoperiod (70 μmol.s^-1^.m^-2^) and a day/night temperature of 23°C/19°C until the plants reached the two leaf stage.

For callogenesis induction, petioles were sectioned (1 cm), longitudinally wounded with a scalpel, and placed in petri dishes containing B5 solid medium supplemented with 1 mg.L^-1^ 2,4-dichlorophenoxyacetic acid. The cultures were maintained at 23°C (16 h) and 19°C (8 h) in the dark. The callus obtained was separated from the petiole and propagated by subculturing every 6 weeks in B5 solid medium (half concentrated macronutrients) supplemented with 0.1 mg.L^-1^ 2,4-dichlorophenoxyacetic acid. Embryogenic cell suspensions in growth regulator-free B5 Gamborg medium were obtained from calli as previously described (Lecomte et al., 2014), with slight modification. The incubation time was reduced to 2 weeks before filtering, and 1 week before transfer in B5 Gamborg medium without growth regulator.

One milliliter of cell suspension was distributed into each well of 12-well cell culture plates (VWR, Radnor, United States), and then 1 mL of treatment solution in growth regulator-free B5 liquid medium was added. The treatment was repeated after 48 h. Treatment solutions were prepared from the lyophilized REs from AUS001, FRA001, FRA017, and ITA002 fungal strains, dried OEs from FRA001, ITA002, and Abra 43 fungal strains and purified aldaulactone. Final concentrations in wells were: REs at 0.2, 1, 5, and 25% of the concentration observed in the fungal culture; OEs at 25 and 100% of the concentration observed in the fungal culture; aldaulactone at 1.25, 5, 12.5, and 50 μg.mL^-1^. REs were resuspended in growth regulator-free B5 liquid medium. OEs and aldaulactone were dissolved in DMSO and then diluted in growth regulator-free B5 liquid medium. Control treatments were: mock treatment, DMSO (0.1%), fungal culture medium RE and OE. DMSO concentrations in the wells were never higher than 0.1%. All treatment solutions were filter sterilized (0.2 μm) and kept at -20°C until use.

### Microscopic Evaluation of Cell Viability and Embryogenesis Ability

Membrane integrity and cell viability were evaluated as previously described (Lecomte et al., 2014) 2 and 7 days after treatment. Briefly, 100 μL of the cell suspensions were stained with 10 μL fluorescein diacetate (0.1 mg.mL^-1^) and 20 μL propidium iodide (1.5 mg.mL^-1^), and incubated in the dark for 5 min. Stained cells were then observed under a fluorescence microscope (Leica DMR HC) equipped for illumination with a 100 W halogen bulb and a Leica L5 filter cube in order to detect green and red fluorescence simultaneously. The microscope was fitted with a digital camera (Qimaging, Retiga 2000R) and monitored using Image Pro Express 6.0 software. Images were acquired at 3 s and 500 ms exposure, a gain of 1 with 1 × 1 binning (1600 pixels × 1200 pixels, 300 pixels per inch), and the images were saved in 24 bit-color TIFF format. For each condition, three microscope slides were prepared from three different cell culture wells and three images were taken per slide. Green and red fluorescence indicated living and dead cells with damaged membranes, respectively. The ability of cells to differentiate and develop somatic embryos was then monitored 3 weeks post-treatment. Proembryogenic masses and somatic embryo formation were visually checked under a stereomicroscope (Olympus SZ61TR).

### Microscopic Image Analysis

Two methods were used: manual and automated fluorescent area evaluation. The toxic effect was estimated by calculating a percentage of living cells from green and red fluorescent areas. First, a manual method based on visual assessments was performed using the GNU Image Manipulation Program (Kimball and Mattis, 2013). The ‘*curves’* tool was used to enhance picture contrast and brightness. Thereafter, the ‘*posteriz*’ tool was used to reduce the number of colors. The suitable posterization level was determined by visual assessment. The ‘*select by color’* tool was then used to select red and green areas and compute the number of green and red pixels in the image. Finally, these numbers were manually recorded in an Excel software file. The whole process was estimated to require several minutes of skilled work per image.

Secondly, a macro-based automated method was implemented. It was performed using ImageJ (Schindelin et al., 2015). We created an ImageJ macro for automatic determination of green and red areas. This macro can be applied in batch and requires about 1 s of machine time per image. 24-bit RGB (red green blue) pictures were converted into composite images. The ‘*split channels’* command was used to separate the three different channels. Green and red channels were then analyzed separately. Green channel data were treated as follows: first, all pixel values above 50 were set at 50 using the ‘*max’* function. Second, all pixel values were multiplied by 4. Then the *‘subtract background’* algorithm was used with the ‘*rolling*’ factor set at 50 pixels. When the picture’s biggest object radius was higher, we used a rolling factor of 100. The processed image was then binarized, with threshold values set at 25 and 255, and saved as a mask. The total green area of objects of more than 30 pixels^2^, was finally determined using the *‘analyze particles’* tool. Red channel data were treated as follows: first, the *‘brightness/contrast’* function was used, with minimum and maximum values set at 18 and 23, respectively. The image was subsequently binarized, with threshold values set at 50 and 255, and further processed using the *‘erode’* tool and then saved as a mask. The total red area of objects of more than 30 pixels^2^, was finally determined using the *‘analyze particles’* tool. Living cell percentages were calculated from green and red areas using Microsoft Excel (2016), and then stored for further analysis.

### Statistical Analysis of Microscopic Images

All statistical analyses were performed using the R v 3.2.4 software in R Studio v 1.0.136 (R Studio Team, 2016). Agreement between the two microscopic image analysis methods was assessed using a Passing-Bablok regression (Bendix et al., 2015). Normality and homoscedasticity of residues were checked by a Shapiro test and Bartlett test, respectively. Toxicity test data were analyzed using a two factor completely randomized design using two plant cell genotypes and 10 treatments as the two factors. Repetitions were treated as blocks. To determine cell viability in relation to the carrot genotype and treatment, data were submitted to multifactorial design ANOVA (type III) using the linear model (Fox and Weisberg, 2017), followed by a Tukey’s HSD [honest significant difference *post hoc* test (de Mendiburu, 2017)]. The normality of the residues was tested using the Shapiro test and their homoscedasticity was tested by graphical assessments.

## Results

### Toxicity of Raw *A. dauci* Exudates on Embryogenic Cells Linked With the Aggressiveness of the Fungus on Whole Plants

In a first round of experiments, we explored a possible correlation between fungal exudate toxicity and fungal aggressiveness. Carrot cell suspensions from three genotypes (resistant I2 and K3, and susceptible H1) were challenged with REs from different *A. dauci* strains at 0.2 to 25% concentrations. The results of these experiments are shown in **Table 1**. Treatments with uninoculated fungal growth medium RE yielded results similar to those of untreated cultures, i.e., regardless of the genetic background, cells survived well after treatment and underwent embryogenesis 3 weeks later. Similar results were obtained using RE from the weakly aggressive AUS001 strain. Whatever the fungal strain, fungal RE did not suppress the embryogenic ability of cells from the resistant I2 and K3 genotypes. Somatic embryo formation from I2 and K3 cell cultures was nevertheless partially affected by the highest RE concentration (25%) from the highly aggressive ITA002 strain. The I2 genotype was also partially affected by 25% RE from the FRA001 strain, but that was not the case for K3. In contrast, 25% RE from ITA002 and FRA001 suppressed the embryogenic ability of cells from the susceptible H1 genotype. Moreover, 5% RE from ITA002 and 25% RE from the moderately aggressive FRA017 strain also affected H1 somatic embryo formation. These results indicated a link between RE toxicity and *A. dauci* aggressiveness.

**Table 1:** Evaluation of raw extract toxicity of differentially aggressive fungal strains on two carrot genotypes.

Medium^a^	Strain	Concentration	Carrot genotype		
			H1	I2	K3
No treatment			++^b^	+++	++

		**25%**^*c*^	**+**	**++**	**+**
	C	5%	+	+++	++
		1%	++	+++	++
		0.2%	++	+++	++
	
		**25%**	**+**	**++**	**+**
	AUS001	5%	+	+++	++
		1%	++	+++	++
		0.2%	++	+++	++
	
		**25%**	**-/+**	**++**	**++**
Carrot juice	FRA017	5%	+	+++	++
		1%	++	+++	++
		0.2%	++	+++	++
	
		**25%**	**-**	**+**	**+**
	FRA001	5%	+	+++	++
		1%	++	+++	++
		0.2%	++	+++	++
	
		**25%**	**-**	**+**	**+/-**
	ITA002	5%	**-**/+	++	++
		1%	+	+++	++
		0.2%	+	+++	++

PDB	C	**25%**	**+**	**+++**	**++**
	FRA017	**25%**	**-**	**++**	**++**

*^a^Fungal culture medium. ^b^The signs are as follows: (-) no embryogenesis was visible and cells were damaged, (+) early-stage embryogenic masses were visible, (++) embryos were present, (+++) embryogenesis was profuse. +/- early-stage embryogenic masses were visible, or no embryogenesis was visible depending on the repetition. The scale is illustrated in Lecomte et al. (2014), Figure 4. ^c^Results obtained with a 25% concentration are in bold character. Carrot cell suspensions with three different genotypes were tested for embryogenesis in the presence of raw extract from A. dauci AUS001, FRA001, FRA017, and ITA002 strains grown in carrot juice medium, and in the presence of raw extract from the A. dauci FRA017 strain grown in potato dextrose broth (PDB). Embryogenesis was assessed 3 weeks after treatment. Cell suspensions either treated with uninoculated media raw extract (C) or untreated were used as an experimental control.*

### *A. dauci* Organic Exudate Production Correlates With Fungal Aggressiveness on Whole Plants

**FIGURE 1:**
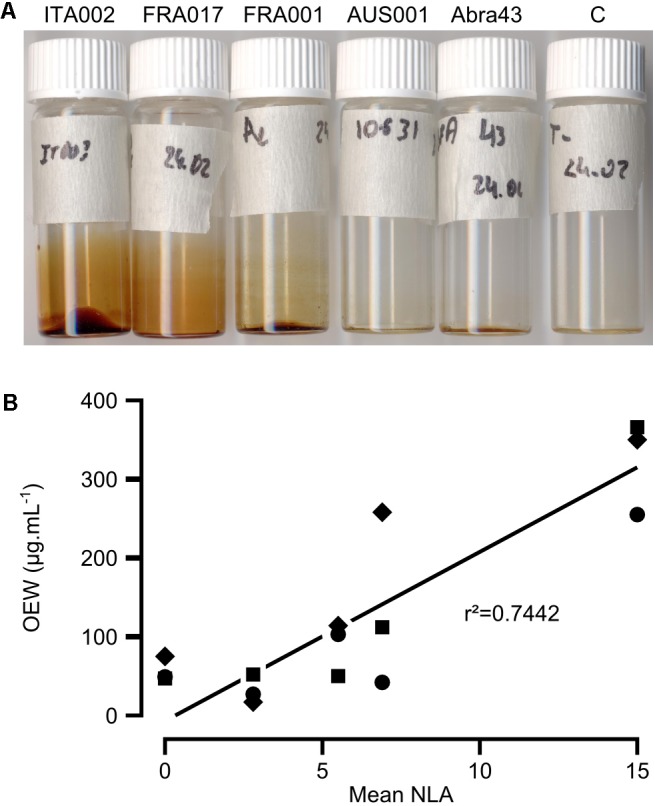
Correlation between aggressiveness and the quantity of organic extracts (OE) amongst *Alternaria dauci* strains. **(A)** OE from liquid cultures of *A. dauci* AUS001, FRA001, FRA017, and ITA002 strains and the *A. brassicicola* Abra 43 strain grown in PDB. OE from the highly aggressive ITA002 strain was more pigmented and looked more abundant, while OE from the weakly aggressive AUS001 strain and the *A. brassicicola* Abra 43 strain showed much less pigmentation. C: OE from uncultured PDB. **(B)** OEW from three different cultures of *A. dauci* AUS001, FRA001, FRA017, and ITA002 strains and of the *A. brassicicola* Abra 43 strain were compared with aggressiveness data obtained previously (mean NLA in Boedo et al., 2012, Table 3, for Abra 43, data not shown). A close correlation coefficient was measured. *Squares*: first repetition, *circles:* second repetition, *diamonds:* third repetition, *r*^2^: correlation coefficient as given in **Supplementary Table S2**.

An OE was derived from the RE to analyze fungal exudates. Interestingly, vials containing OE looked very different depending of the strain (**Figure 1A**). Indeed, we found marked variations in the quantity of OE depending on the fungal strain. Typical yields (±SE) were 57 ± 11 μg.mL^-1^ for the *A. brassicicola* Abra 43 strain (non-pathogenic on carrot), 32 ± 13 μg.mL^-1^ for the weakly aggressive AUS001 strain, 89 ± 24 μg.mL^-1^ and 137 ± 78 μg.mL^-1^ for the two intermediate FRA017 and FRA001 strains, and 324 ± 42 μg.mL^-1^ for the highly aggressive ITA002 strain. The aggressiveness data reported by Boedo et al. (2012) and the OEW data obtained here did show a significant correlation when plotted against each other (*r*^2^ = 0.7442, **Figure 1B**).

### Organic Compound Production by *A. dauci* Closely Correlates With Fungal Aggressiveness on Whole Plants

**FIGURE 2:**
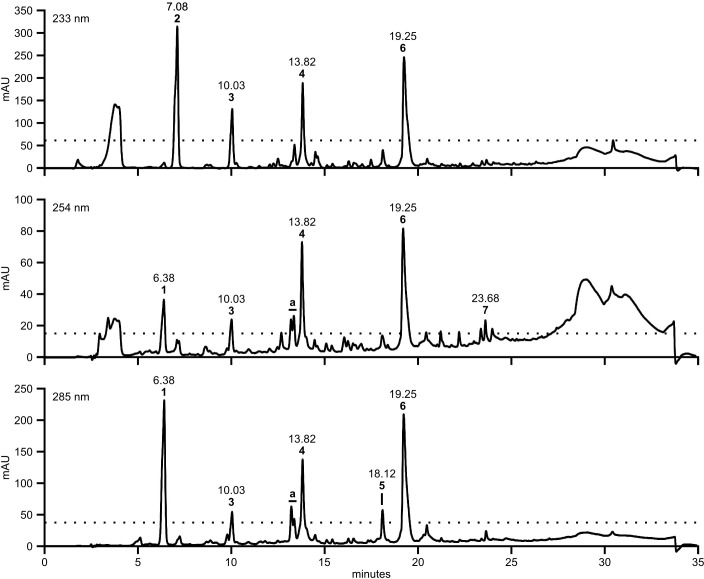
A large number of UV active compounds are present in *A. dauci* exudates. Organic extracts from *A. dauci* FRA001 strain liquid cultures were analyzed using HPLC-DAD. Absorption profiles revealed a complex pattern, with numerous peaks. Major peaks were selected as follows: at each wavelength, a threshold, corresponding to 20% absorption observed at the highest peak, was defined (dotted line). Seven clearly defined peaks (numbered 1–7 in bold figures) with a maximum absorption clearly above this threshold for at least one wavelength were taken into consideration for further study. The bold letter a denote peaks that were higher than the threshold, but not well separated. The retention times shown here correspond to the presented sample. Mean retention times with standard errors are presented in **Table 2**.

**FIGURE 3:**
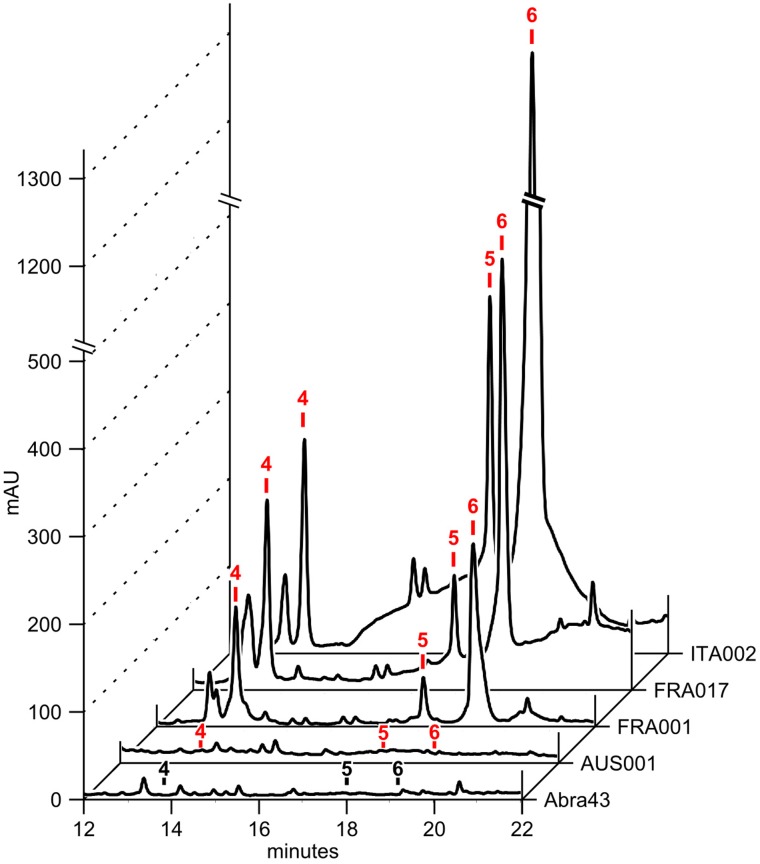
Exudates from different *A. dauci* strains have a variable composition. 50 μg of OE from *A. dauci* AUS001, FRA001, FRA017, and ITA002 strains and *A. brassicicola* Abra 43 strain liquid cultures were analyzed using HPLC-DAD. The absorption profiles shown here at 285 nm revealed complex patterns that varied from strain to strain. Major peak numbers, as reported in **Table 2**, are indicated at the corresponding retention time with a black label when the peak is absent or a red label when present.

HPLC-DAD analysis of OEs from three different cultures of *A. dauci* AUS001, FRA001, FRA017, and ITA002 strains and the *A. brassicicola* Abra 43 strain revealed complex patterns with numerous peaks (**Figure 2**) and high variation in composition amongst strains (**Figure 3**). Seven high peaks were repeatedly observed in OEs from several different strains. Peaks were numbered from 1 to 7 depending on the retention time (**Table 2**). AUC calculated for each peak at three wavelengths: 233 (a), 254 (b) and 285 (c) nm, and data correlations (*r*) were computed for each OE. For peaks 4, 6, and 7, *r* was above 0.9 for each wavelength pair, and a mean AUC (*m*) was computed for each analyzed OE. Peaks 1, 2, 3, and 5 did not meet this criterion. The minimal *r*-value ranged from 0.436 (peak 3) to 0.580 (peak 1), and 0.623 (peak 5), except for peak 2 where a strongly negative *r* (-0.953) was observed. For these peaks, AUC values corresponding to the two least correlated wavelengths were analyzed separately.

**Table 2:** HPLC analysis of fungal organic extracts: major peaks.

Peak n°	Retention time (minutes)	Correlation coefficients (*r*)		
		233–254	233–285	254–285
1	6.42 ± 0.01	**0.743^a^**	**0.580^b^**	**0.899**
2	7.16 ± 0.01	**0.860**	**-0.953**	**-0.565**
3	10.09 ± 0.01	**0.508**	0.992	**0.436**
4	13.79 ± 0.01	0.953	0.988	0.945
5	17.98 ± 0.01	**0.623**	0.947	**0.728**
6	19.13 ± 0.06	0.998	0.994	0.995
7	23.72 ± 0.01	0.997	0.994	0.999

*^a^r-values below 0.9 are in bold numbers. ^b^Underlined r compare peaks considered to correspond to different compounds. Organic extracts from three different cultures of A. dauci AUS001, FRA001, FRA017, and ITA002 strains and the A. brassicicola Abra 43 strain were analyzed using HPLC-DAD. Seven major peaks were selected for area under a curve statistical analysis. For each peak, retention time (RT) and AUC were measured for three light wavelengths: 233, 254, and 285 nm. Correlation coefficients (r) were computed for each wavelength pair. Each peak was considered to correspond to a single molecule when r was above 0.9.*

**FIGURE 4:**
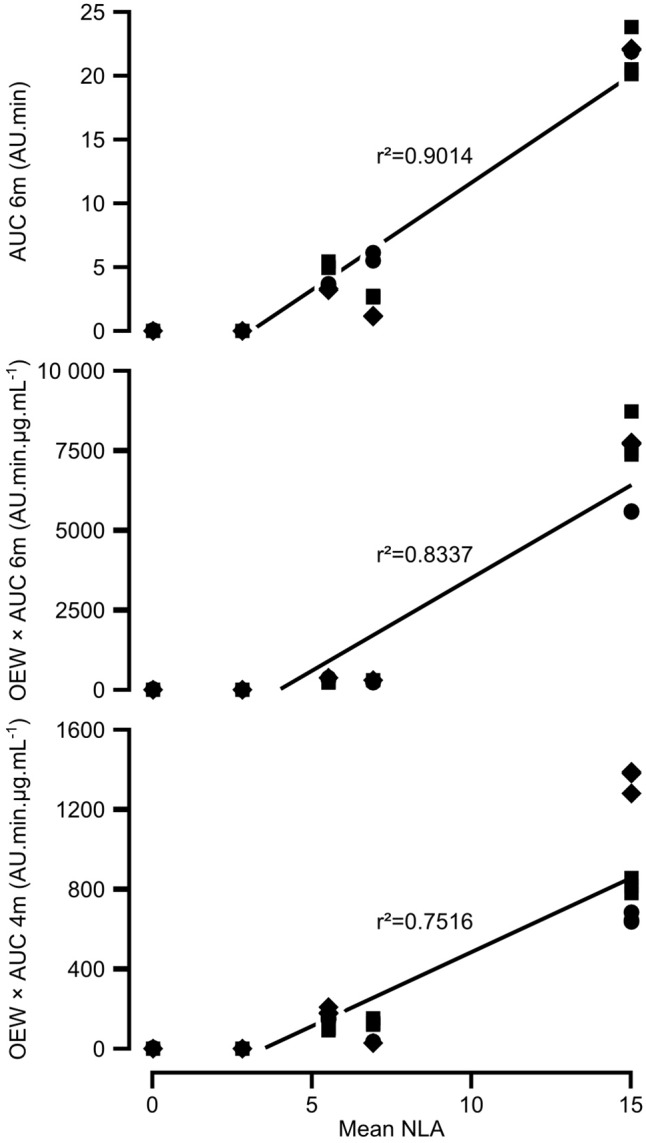
Correlations between aggressiveness and the composition of organic extract amongst *A. dauci* strains. Organic extracts from three different cultures of each *A. dauci* AUS001, FRA001, FRA017, and ITA002 strains and the *A. brassicicola* Abra 43 strain were analyzed using HPLC-DAD. Eleven candidate compounds were selected for area under a curve (AUC) statistical analysis from seven major peaks, as shown in **Table 2**. Organic extract weight (OEW), and AUC and OEW × AUC for each candidate compound were used as variables in 29 linear models of aggressiveness based on data obtained previously [mean NLA in Boedo et al. (2012), Table 3]. Three variables, corresponding to peaks 4 and 6, showed a closer correlation with aggressiveness than OEW: OEW × AUC 4m, OEW × AUC 6m, and AUC 6m (see **Supplementary Table S2**). AUC 4m and AUC 6m are linear means of AUC for peaks 4 and 6, respectively, calculated at 233, 254, and 285 nm. OEW × AUC 4m, OEW × AUC 6m, and AUC 6m raw data are plotted against strain aggressiveness. *Squares*: first repetition, *circles:* second repetition, *diamonds:* third repetition, *r*^2^: correlation coefficient as given in **Supplementary Table S2**.

**FIGURE 5:**
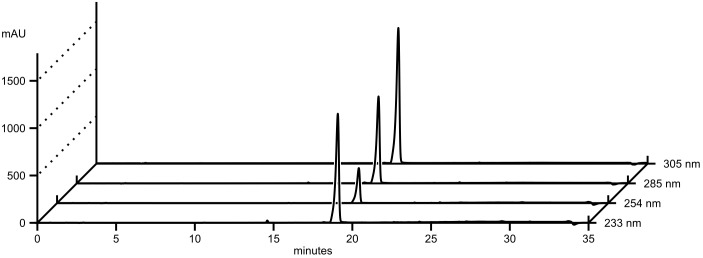
HPLC-DAD analysis of purified aldaulactone. The compound corresponding to peak 6 in **Figure 2** was purified using flash chromatography. Absorbance data are shown at four wavelengths.

Comparison of AUC and AUC × OEW data with aggressiveness data from (Boedo et al., 2012) revealed that AUC × OEW was significantly correlated with aggressiveness for all analyzed peaks except 1c, 2c, 3c, and 7m. Conversely, AUC was only correlated with aggressiveness for peaks 1c, 4m, 5c, and 6m. This substantial difference could be explained by the fact that OEW was closely correlated with aggressiveness. Interestingly, AUC data for peak 6m and AUC × OEW data for peaks 4m and 6m showed a closer correlation with aggressiveness than OEW (**Figure 4** and **Supplementary Table S2**). Moreover, for peak 6m, AUC was more correlated with aggressiveness than AUC × OEW. The compound corresponding to peak 6m was thus considered as a good potentially toxic candidate, and was purified (**Figure 5**) and further analyzed.

### Aldaulactone Purification and Characterization

**FIGURE 6:**
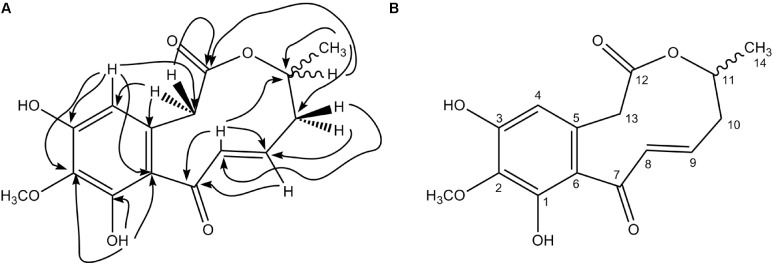
Aldaulactone structure. **(A)** HMBC correlations for aldaulactone; **(B)** structure numbering.

Aldaulactone was isolated as a light yellow amorphous solid from the ethyl acetate extract of *A. dauci* (strain ITA002) cultures. This compound has maximum absorbance of 305 nm. Its chemical ionization high-resolution mass spectrum analysis revealed a pseudo-molecular ion with C_15_H_17_O_6_ with the following formula: *m/z* 293.1028 [M+H]^+^, (calculated *m/z* 293.1020). The ^1^H NMR spectrum recorded in acetone-*d_6_* highlighted the presence of a signal at δ_H_ = 13.90 ppm typical of a chelated phenolic proton. The ^13^C NMR spectra showed 15 signals with two downfield signals corresponding to carbonyl groups at δ_C_ = 199.4 and 173.0 ppm. HMQC (**Figure 6A**) highlighted the presence of two methylene carbons at δ_C_ = 42.0 and 44.1 ppm bearing two protons each at δ_H_ = 2.17/2.58 ppm (respectively, dd and ddd) and 3.73/4.06 ppm (respectively, d and dd) with a 17.3 Hz *gem* coupling constant. The position of the latter methylene group (H_13a_ and H_13b_) between the aryl group and the lactone carbonyl function was confirmed by heteronuclear multiple bond correlation (HMBC) cross-peaks from H_4_ to C_13_ and from H_13a/b_ to C_12_. Furthermore, the lactone carbonyl carbon showed a correlation with H_11_. The HMQC correlation confirmed that this proton is attached to a methine carbon at δ_C_ = 74.6 ppm and directly connected to the endocyclic oxygen atom of the lactone ring. The ^1^H NMR spectrum highlighted the presence of a methyl doublet (δ 1.38, *J* = 6.4 Hz) also attached to the C_11_ methine carbon. Cross-peaks from H_14_ to the second methylene carbon C_10_ and from H_10a/10b_ to C_14_ were also observed in the HMBC spectrum. Based on HMQC correlations, vinylic protons were also identified at δ_H_ = 5.99 (ddd) and 6.86 ppm (d, *J* = 16.3 Hz) with the corresponding sp^2^ carbons at δ_C_ = 138.5 and 136.0 ppm, respectively. The HMBC spectrum showed correlations for these protons with the carbon of a carbonyl group at δ_C_ = 199.4 ppm, characteristic of an α,β-unsaturated ketone moiety. Correlations observed in the HMBC spectrum between the vinylic protons H_8_/H_9_ and the methylene carbon C_10_ allowed us to define the aldaulactone backbone as shown in **Figure 6A**. This compound is a new member of the 10-membered benzenediol lactone molecule family. Chemical shifts are detailed in **Table 3** associated with the structure numbered (**Figure 6B**).

**Table 3:** ^1^H (400 MHz, Acetone-*d_6_*) and ^13^C NMR (100 MHz, Acetone-*d_6_*) spectroscopic data for aldaulactone.

Position	δ_H_(ppm), coupling (*J*, Hz)	δ_C_ (ppm)
1	-	160.7
2	-	134.1
3	-	155.4
4	6.40, s	113.8
5	-	135.2
6	-	112.8
7	-	199.4
8	6.86, d (16.3)	136.0
9	5.99, ddd (16.3, 10.5, 5.7)	138.5
10	2.17, dd (22.9, 10.5)	42.0
	2.58, ddd (12.0, 5.7, 2.3)	
11	5.10–5.32, m	74.6
12	-	173.0
13	3.73, d (17.3)	44.1
	4.06, dd (17.3, 1.0)	
14	1.38, d (6.4)	19.2
1-OH	13.90, s	-
2-OCH_3_	3.78, s	59.6
3-OH	9.05, bs	-

### Aldaulactone Quantification in Fungal Exudates

Aldaulactone contents of OEs were determined using an external standard HPLC method. Three biological replicates of Abra 43 strain OE were analyzed by HPLC at 305 nm. No peak was detected at the aldaulactone retention time (19.13 ± 0.06 min), suggesting an absence of significant amounts of the compound (<0.102 mg.mL^-1^) in Abra 43 OE. Four biological replicates of ITA002 and FRA001 strain OEs were also analyzed by HPLC at 305 nm light wavelength. FRA001 OE and ITA002 OE contained 7.9 and 21% of aldaulactone, respectively. From these results, aldaulactone concentrations in FRA001 and ITA002 REs were determined at 5.1 ± 4.3 μg.mL^-1^ and 42.8 ± 26.4 μg.mL^-1^, respectively (mean ± CI). The aldaulactone concentration in ITA002 culture is significantly greater than the aldaulactone concentration in FRA001 culture. These results validated previous observations based on the OE HPLC profiles.

### Aldaulactone Toxicity Partly Explains OE Toxicity

**FIGURE 7:**
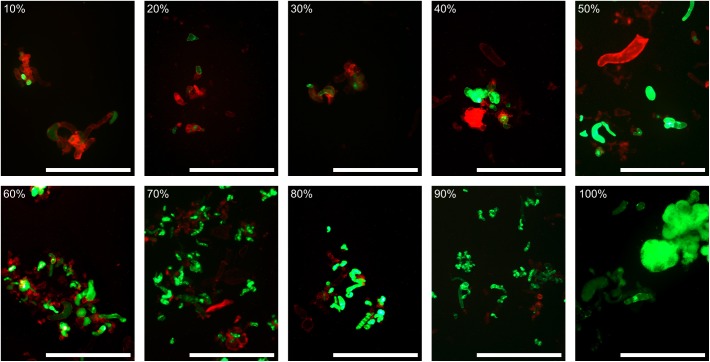
A scale of automatically calculated live cells percentage. During this study, more than 550 fluorescein diacetate/propidium iodide stained carrot cell images were obtained. For each image, a percentage of live cells was calculated using our macro-based automated method. For values from 10 to 100% live cells, representative images were selected. Effective percentage of live cells is within one percent of the scale value. Red channel and contrast were then normalized for visibility. Bar: 1 mm.

We performed toxicity test experiments to compare aldaulactone and OE toxicities. Liquid cell cultures from the two carrot H1 and I2 genotypes were challenged by adding nine different treatments to the plant cell culture medium. Microscopic macro-based evaluation of cell viability was achieved 7 days after treatment and embryogenesis ability was scored 3 weeks after treatment. In order to assess our new live and dead cell automated quantification method, we used a set of 97 microscopic images from one of our three toxicity assays. This image set was processed by visual assessment- and macro-based image analysis methods. Two datasets containing measured living cell percentages were thus generated. To assess the macro-based image analysis method, a Passing-Bablok regression analysis was performed on these two datasets. The confidence interval of 95% around the intercept and the slope, respectively, contained the values zero and one. We thus accepted the null hypothesis, i.e., there was no constant or proportional difference between the two methods. The automated method selects pixels the same way as the conventional visual-assessment method. We thus decided to perform this macro-based method on all the microscopic images obtained from the three toxicity test experiments. Images corresponding to 10, 20, …, 100% of living cells were selected for illustration (**Figure 7**). ANOVA analysis showed a significant interaction between genotype and treatment factors corresponding to the stronger resistance of I2 cells to treatments. Control conditions (control, DMSO and medium OE) and OE from the non-pathogenic Abra 43 strain gave results in the same homogeneity groups for both H1 (a) and I2 (d and e) genotypes (**Table 4**). As expected, after OE treatment of FRA001 and ITA002, the mean percentage of living cells in H1 was significantly lower. FRA001 and ITA002 strain OEs induced a 55% reduction in H1 cell viability compared to medium OE. Aldaulactone concentrations of 12.5 and 50 μg.mL^-1^ significantly decreased the cell viability (-30.5%), thus confirming the toxicity of the compound. There was no significant effect of FRA001 OE and all aldaulactone concentration treatments on I2 cell viability. Only ITA002 OE had a significant effect on I2 cells, with a 23.5% reduction compared to medium OE.

**Table 4:** Influence of carrot genotype, fungal organic extract and aldaulactone treatments on carrot cell suspension viability.

Carrot genotype	Treatment^a^	Mean percentage live cells	Homogeneity groups^b^
H1	Control	75.6	ab
	DMSO 0.1%	75.0	ab
	Medium OE	83.9	a
	Abra 43 OE	64.3	abcd
	FRA001 OE	29.8	ghi
	ITA002 OE	29.0	hi
	Aldaulactone 1.25 μg.mL^-1^	76.4	ab
	Aldaulactone 5 μg.mL^-1^	74.4	abc
	Aldaulactone 12.5 μg.mL^-1^	53.4	def
	Aldaulactone 50 μg.mL^-1^	52.6	def

I2	Control	57.3	bcde
	DMSO 0.1%	55.3	cdef
	Medium OE	50.0	defg
	Abra 43 OE	49.4	defgh
	FRA001 OE	36.2	fghi
	ITA002 OE	26.5	i
	Aldaulactone 1.25 μg/mL	51.4	def
	Aldaulactone 5 μg/mL	48.9	defgh
	Aldaulactone 12.5 μg/mL	51.3	def
	Aldaulactone 50 μg/mL	40.6	efghi

*^a^The treatments were applied as follows: Control: untreated cells; DMSO: a DMSO solution at 0.1%, the DMSO concentration used to prepare OE and aldaulactone solutions; Medium OE: organic extract of fungal culture medium; Abra 43 OE: A. brassicicola strain Abra 43 fungal culture organic extract; FRA001 OE: A. dauci strain FRA001 fungal culture organic extract; ITA002 OE: A. dauci strain ITA002 fungal culture organic extract. ^b^Homogeneity groups were obtained through a type III ANOVA analysis of three independent biological repetitions, followed by a Tukey’s HSD post hoc test. When the same letters were present for two genotype-treatment pairs, they are not significantly different. Carrot cell suspensions of susceptible (H1) and partially resistant (I2) genotypes were challenged with fungal organic extract and the purified toxin candidate aldaulactone. Cell viability was evaluated by a macro-based image analysis of nine images of double stain fluorescein diacetate/propidium cells for each condition*.

To assess this result and further analyze the treatment toxicity, the cell ability to differentiate and develop somatic embryos was monitored according to visual scores 3 weeks after treatment using the scale proposed in Lecomte et al. (2014). For all control conditions and both genotypes (**Table 5**), we observed embryos, numerous embryogenic masses and many living cells. The embryo density in I2 cultures was lower than in H1. I2 cell cultures treated by Abra 43 OE showed the same response as untreated cultures, while H1 cultures contained slightly fewer embryos. H1 and I2 cultures treated with FRA001 OE both presented no embryo development and some cell debris but also a number of living cells and embryogenic masses. Compared with control conditions, H1 cells were thus more affected by FRA001 OE than I2 cells. ITA002 OE had a stronger effect on H1 cells than FRA001, i.e., much cell debris, and only a few embryogenic masses were observed. I2 cultures treated with ITA002 OE showed no embryos and some cell debris but also a quantity of living cells and embryogenic masses like those treated with FRA001. Aldaulactone presented a dose-dependent phytotoxic activity on H1 cells, from a slight effect on embryogenesis visible at 1.25 μg.mL^-1^ to a strong effect at 50 μg.mL^-1^. This effect was only slightly visible in I2 cultures at the highest aldaulactone concentration tested (50 μg.mL^-1^). These results confirmed the cell viability assessment data.

**Table 5:** Influence of carrot genotype, fungal organic extract and aldaulactone treatments on carrot cell embryogenesis aptitude.

Carrot genotype	Treatment^a^	Evaluation of somatic embryogenesis^b^
H1	Control	+++
	DMSO 0.1%	+++
	Medium OE	+++
	Abra 43 OE	++
	FRA001 OE	+
	ITA002 OE	+/-
	Aldaulactone 1.25 μg.mL^-1^	++
	Aldaulactone 5 μg.mL^-1^	+
	Aldaulactone 12.5 μg.mL^-1^	+
	Aldaulactone 50 μg.mL^-1^	+/-

I2	Control	++
	DMSO 0.1%	++
	Medium OE	++
	Abra 43 OE	++
	FRA001 OE	+
	ITA002 OE	+
	Aldaulactone 1.25 μg.mL^-1^	++
	Aldaulactone 5 μg.mL^-1^	++
	Aldaulactone 12.5 μg.mL^-1^	++
	Aldaulactone 50 μg.mL^-1^	+

## Discussion

The present study aimed to explore the link between *A. dauci* aggressiveness and toxin production. In our previous study (Lecomte et al., 2014), a close correlation between the resistance of whole carrot plants to *A. dauci* and carrot cell resistance to the toxic organic phase of fungal exudates was shown. Here a correlation between fungal aggressiveness and fungal exudate toxicity on *in vitro* cultured carrot cells was obtained. We hypothesized that the toxicity of the fungal exudates was correlated with the amount of toxic compounds produced by the fungus. A link between the raw exudate or OE toxicity on carrot cells and *A. dauci* aggressiveness was then shown. The fungal exudate OEs were analyzed using HPLC-DAD techniques and a correlation between the quantitative production of organic metabolites and the aggressiveness of the fungal strains was established. The analysis of the different HPLC spectra revealed that the presence of one specific peak was strongly correlated with fungal aggressiveness. A closer aggressiveness correlation was found with the area under a curve for this specific peak as compared with the weight of OE obtained from exudates. NMR analysis and a purification step confirmed that this peak corresponded to a single molecule. Further structural studies using high-resolution mass spectrometry or bidimensional NMR analyses allowed the characterization of an original 10-membered benzenediol lactone that we named aldaulactone. This metabolite had a structure very similar to those of xestodecalactones D-F isolated from a *C. cassiicola* strain that in turn was isolated from *Laguncularia racemosa* (Ebrahim et al., 2010). The aldaulactone backbone was also closely correlated with sporostatin, a molecule purified from *Sporormiella* sp. M5032 (Kinoshita et al., 1997).

A new cell viability assessment based protocol was developed to evaluate the toxicity of OEs and aldaulactone. In a previous study, Lecomte et al. (2014) used a fluorimetric method for measuring esterase activity using fluorescein diacetate as substrate. However, this method was time-consuming and more suitable for evaluating carrot cell resistance than for studying the effects of a toxin on cell viability. Here we adapted the double stained protocol of Vitecek et al. (2007) and developed an automated macro-based relative evaluation of living cell percentages. The efficiency of the automated image analysis method was confirmed by comparing it with expert visual assessments. This macro allowed fast assessment of hundreds of images without experimenter bias, and was also much less time consuming than the enzymatic assay mentioned above.

Quantitative cell viability evaluations were supplemented with more qualitative embryogenesis assessments. Both toxicity evaluations showed a toxic effect of *A. dauci* OE. Unlike the H1 culture response, I2 cultures were only slowly and weakly affected by fungal OE. This confirmed the already observed correlation between plant pathogen resistance and plant cell resistance toward fungal extracts (Lecomte et al., 2014). Moreover, the stronger aggressiveness of ITA002 compared to FRA001 paralleled the significantly different viability and embryogenic availability of I2 cells treated with ITA002 and FRA001 OEs. These results were generally in line with previous observations of differential resistance of H1 and I2 cells to *A. dauci* FRA017 OE toxicity (Lecomte et al., 2014). They also suggest that this differential resistance is not strain dependant. Both criteria also indicated that aldaulactone was toxic to carrot cells. Like FRA001 and ITA002 OEs, aldaulactone induced a delay in embryonic development and a decrease in cell viability. Moreover, H1 susceptibility and I2 resistance to OE could be paralleled with their resistance and susceptibility to aldaulactone. This dataset showed that aldaulactone toxicity is a key component of fungal exudate toxicity, and that aldaulactone is involved in both fungal pathogenicity and plant resistance mechanisms. Nevertheless, the higher toxicity of OEs as compared to that of corresponding aldaulactone concentrations indicated that co-factors or other toxins were perhaps also involved in these mechanisms.

### Toxin Production by Phytopathogenic Fungi and Links With Plant Resistance or Fungal Diversity

Numerous fungal phytotoxins are described in the literature as they play key roles in fungal pathogenicity mechanisms, especially amongst necrotrophs, including the *Alternaria* genus. In many cases, toxins were studied for their interactions with plants (non-host resistance and varietal resistance) or for their biosynthesis by various fungi. Few studies have considered both partners of the studied pathosystem. A link between *Alternaria* sp. aggressivity on tomato and abundance of tenuazonic acid, alternariol and alternariol monomethyl ether in liquid culture filtrates has been suggested by Meena et al. (2017b). Tenuazonic acid, alternariol and alternariol monomethyl ether, have also been shown to induce hydrogen peroxide accumulation in tomato leaves (Meena et al., 2016). Toxins, such as the SS toxin from *S. solani* culture filtrates, causing garlic leaf blight, exhibit different levels of phytotoxicity to a range of hosts (Zheng et al., 2009). Viridiol, produced by *Hymenoscyphus pseudoalbidus*, was identified as a phytotoxin responsible for necrotic activity against ash seedlings (Andersson et al., 2010). The use of high concentrations of the phytotoxin was helpful in detecting ash genotypes resistant to the fungal pathogen by comparison to susceptible ones (Cleary et al., 2014).

Phytotoxin production by different fungal strains was evaluated in other studies. The *in vitro* phytotoxic effects of culture filtrates from three *Fusarium oxysporum* strains were tested on vanilla plantlets and were correlated with the production of fusaric acid (Ramírez-Mosqueda et al., 2015). Analysis of OEs from the cultures of 13 isolates of *Neofusicoccum parvum*, i.e., the main causal agent of *Botryosphaeria* dieback in grapevine, revealed similar metabolite patterns among isolates. Using one isolate, 13 metabolites belonging to four different chemical families were isolated and characterized, while two phytotoxins were characterized as epoxylactones (Abou-Mansour et al., 2015).

Only a few studies have been specifically based on the involvement of toxins in pathogenicity and mechanisms of plant partial resistance responses. Among them is the study of Zheng et al. (2010) on the non-proteinaceous SS toxin from *S. solani*, along with another key study focused on the *C. cassiicola*-rubber tree pathosystem. *C. cassiicola* produces a small cysteine-rich glycoprotein named cassiicolin, which was identified as a potential disease effector (Breton et al., 2000; Barthe et al., 2007; de Lamotte et al., 2007). Cassiicolin isolation was achieved from two isolates with high and medium aggressiveness, but not from a less-aggressive isolate, while the difference in isolate aggressiveness was correlated with variations in cassiicolin transcript levels. A fungal diversity study based on the same pathosystem showed that isolates carrying the *Cas 1* gene, which encodes cassiicolin, were the most aggressive isolates on two rubber tree cultivars (Déon et al., 2014). Although the present study was not based on a phytotoxic protein, our results highly suggest that the aggressiveness diversity in *A. dauci* could also be correlated with toxin production in a quantitative manner. The different studies reported above for various pathosystems highlight the very high diversity of toxins in terms of their chemical structures and production depending on the type of interaction with the plant and of course the fungal strain genotype.

### Toxin Production by *Alternaria* sp. With a Focus on *A. dauci*

Different types of metabolites are involved in the interaction between *Alternaria* sp. and plants. These metabolites have been identified as proteins, glycoproteins, saccharides or secondary metabolites (for reviews, see Lou et al., 2013; Meena et al., 2017a). For instance, in exudates collected from *A. brassicicola* germinated conidia, a 1.3 kDa protein called the AB toxin was identified and further characterized as a host-specific toxin (Otani et al., 1998; Oka et al., 2005). To our knowledge, no proteins or saccharides with phytotoxic activity have been identified in *A. dauci*. Moreover, the aqueous phase of *A. dauci* exudates was not found to be toxic in carrot cells (Lecomte et al., 2014). Fungi belonging to the *Alternaria* genus are known to produce more than 250 described low molecular weight secondary metabolites, with many of them toxic to plants (Lou et al., 2013). Several metabolites are unique to a single *Alternaria* species, but most metabolites, such as alternariol, are produced by more than one species. These metabolites are very diverse in their chemical structure, including nitrogen-containing metabolites, steroids, terpenoids, pyranones, quinones, and phenolics (Lou et al., 2013). Several lactone bearing polyketides have already been isolated from *Alternaria* species. The size of the lactone cycle varies from seven centers for alterlactone (Aly et al., 2008) to 12 centers for curvularin derivatives from *A. tomato* and others (Hyeon et al., 1976), and even 13 centers for brefeldin analogs isolated from *A. carthami* and *A. zinniae* (Tietjen et al., 1983; Vurro et al., 1998). Aldaulactone, as the first 10-membered benzenediol lactone identified from *Alternaria* species, exhibits a chemical structure that differs from those mentioned above.

Among the huge diversity of toxic molecules in the *Alternaria* genus, some are described as being involved in host specificity. At least nine diseases caused by *Alternaria* species in which host-specific toxins are responsible for fungal pathogenicity have been reported (Lou et al., 2013). The biosynthesis of brassicicolin A, a major host-specific toxin produced by the black spot agent in Brassicaceae (*A. brassicicola*), was recently described (Pedras and Park, 2016). Nevertheless, the involvement of such host-specific toxins in the *A. dauci*–*D. carota* interaction has never been published to our knowledge.

*Alternaria dauci* strains have been reported to produce numerous toxins, including zinniol (Barash et al., 1981), alternariol monomethyl ether (Montemurro and Visconti, 1992) and several specific uncharacterized secondary metabolites (Andersen et al., 2008). Among these toxins, some have been more particularly studied and well documented. Alternariol monomethyl ether is an important mycotoxin with cytotoxic, genotoxic, and teratogenic effects on mammalians (Bensassi et al., 2011). Zinniol was first described as a non-host specific toxin and has been thought to be involved in *Alternaria* sp. pathogenicity in plants. More recent work contradicts this idea with regard to *A. tagetica* (Qui et al., 2010) and *A. dauci* (Lecomte et al., 2014). Aldaulactone may be considered as a crucial toxic determinant of pathogenicity in the *A. dauci–D. carota* interaction. Further studies will be necessary: (i) to determine if the aldaulactone toxic effect is host-specific or not, and (ii) to highlight pathogenic determinants in *A. dauci* using genomic and transcriptomic approaches.

### Aldaulactone Characteristics: Chemical Structure and Putative Biological Function

Among the huge number of natural secondary metabolites, aldaulactone, a 10-membered benzenediol lactone, belongs to the polyketides family. The polyketides group numerous compounds, which are remarkable by their structural diversity and various biological activities, including phytotoxic, anti-tumoral, antifungal, and antibacterial effects. Among them, 10-membered lactones often acts as phytotoxins and are also associated with various pharmacological activities (Sun et al., 2012). Sun et al. (2012) inventoried 63 10-membered lactones of natural origin that were discovered between 1997 and July 2011. Since that time, to our knowledge, only a few natural 10-membered lactones have been purified and identified, such as cremenolide produced by *Trichoderma cremeum* (Vinale et al., 2016), or cytospolides A-E and F-Q produced by *Cytospora* sp. (Lu et al., 2011a,b). All the macrolactones mentioned above are produced by microorganisms, most of them by fungi. According to Sun et al. (2012), 10-membered lactones are divided into three classes depending on the complexity of the heterocyclic backbone substitution pattern: (i) with only methyl and oxygen susbstituents; (ii) with extended alkyl chains; and (iii) with additional rings. The latter class contains numerous structurally complex derivatives that are also divided into different families. These include benzenediol lactones, i.e., a growing class of metabolites bearing a 1,3-benzenediol moiety bridged by the macrocyclic lactone ring, whose size generally ranges from 8 to 14 centers (Shen et al., 2015). In most described cases, their synthesis requires two type I polyketide synthases, a highly reducing one and then a non-reducing one (Shen et al., 2015). Aldaulactone is a new 10-membered benzenediol lactone. So far only 10 other metabolites with a similar structure have been isolated and characterized from fungi. Aldaulactone is structurally related to sporostatin for its macrolactone ring (**Supplementary Figure S1A**) and xestodecalactone D for its benzene ring (**Supplementary Figure S1B**). Sporostatin was found to be an inhibitor of cyclic adenosine 3′,5′-monophosphate phosphodiesterase (Kinoshita et al., 1997) and a specific inhibitor of epidermal growth factor receptor tyrosine kinase *in vitro* (Murakami et al., 1999). The biological function of aldaulactone has not yet been defined and should be studied further.

## Conclusion and Perspectives

The present results highly suggest that aldaulactone play a role in several aspects of the *D. carota*–*A. dauci* interaction, including the variation in aggressiveness amongst fungal strains and quantitative plant resistance mechanisms. The pathosystem studied here represents a good model to elucidate plant partial resistance mechanisms against a fungal pathogen toxin. Aldaulactone was shown to mimic the OE toxicity of fungal exudates, but its toxicity level was weaker than that of the OE. Moreover, aldaulactone was mainly present in the exudate from the most aggressive strain studied here (ITA002 strain). Based on HPLC-UV analyses, the metabolite profiles showed high qualitative and quantitative variations depending on the strain. This result strongly suggests that aldaulactone could be one of the major toxins produced by *A. dauci*. The interaction between aldaulactone and one or several other molecules may contribute—in an additive or synergistic manner—to the level of toxicity.

The production and/or chemical synthesis of pure aldaulactone in sufficient quantities will be necessary to explore its mode of action and also to assess potential applications with this 10-membered benzenediol lactone. Production of aldaulactone analogs through various semi-synthetic structural modifications, associated with biological evaluation of their toxicity on carrot cells cultures, will be a way to further characterize and understand the mode of action of this new toxin. These 10-membered benzenediol lactone analogs could also be evaluated to explore other biological activities (antibacterial, antifungal, antitumoral, etc.). Potential applications of other *Alternaria* sp. metabolites, as antitumor agents, herbicides or antimicrobials, have already been reported by Lou et al. (2013). Total synthesis of decalactones were recently reviewed by Sun et al. (2012) with different objectives, including medical uses of decalactones or biocontrol applications. On the fungal side, further experiments are needed to identify the genes encoding biosynthetic enzymes responsible for aldaulactone production and to study transcriptional variations in relevant genes among *A. dauci* isolates. On the plant side, partial resistance mechanisms in response to the toxin remain to be clarified, but the availability of purified aldaulactone should facilitate this task. Aldaulactone plant cellular targets may be studied in future experiments. Further assays are needed to determine if aldaulactone is a host-specific toxin or not. In order to highlight the differential mechanisms in resistant vs. susceptible carrot genotypes, the plant response to the phytotoxin may be analyzed through omic approaches, as previously described for ash infected by viridiol (Cleary et al., 2014). From a more practical standpoint, the selection of carrot genotypes resistant to aldaulactone could be performed using the imagery method described here and perhaps used in the future by breeders to improve carrot resistance to *A. dauci*.

## Author Contributions

RB and PP: designed the project. RB, PP, SG, LH, and J-JH: managed the project. RB, PP, JC, LH, and J-JH: wrote the article. TB: contributed to the drafting and the critical revision of the article. MB, ML, JC, and BH: contributed the fungal and plant materials. JC, J-JH, ML, YR, and EG: produced, extracted, and analyzed the fungal extracts. J-JH, YR, and SG: purified and characterized the aldaulactone. JC, LH, ML, LV, LO, and CY: performed the plant *in vitro* experiments. JC, LH, CY, TB, and RB: performed the image and data analysis. J-JH, JC, RB, and PP: edited the article. All authors approved the final version of the manuscript.

## Conflict of Interest Statement

The authors declare that the research was conducted in the absence of any commercial or financial relationships that could be construed as a potential conflict of interest.

## Supplementary Material

FIGURE S1 Compounds structurally related to aldaulactone. **(A)** Sporostatin; **(B)** xestodecalactone D.

TABLE S1 List of characterized genes corresponding to quantitative disease resistance loci. This table sums up literature presented previously (Poland et al., 2009; Lecomte et al., 2014; French et al., 2016) or not (for *At5g22540*, *qPLSr5a*, and *pan1*). Mixed-up references presented in (French et al., 2016) have been sorted out.

TABLE S2 HPLC analysis of fungal organic extracts: correlations with aggressiveness. Organic extract from three different cultures of *A. dauci* AUS001, FRA001, FRA017, and ITA002 strains and the *A. brassicicola* Abra 43 strain were analyzed using HPLC-DAD. Seven major peaks were selected for area under a curve (AUC) statistical analysis. Eleven candidate compounds were derived from those peaks, as described in the section “Materials and Methods.” OEW, and AUC and OEW × AUC for each candidate compound were used as variables in 23 linear models of aggressiveness based on data obtained previously [mean NLA in Boedo et al. (2012), Table 3]. For each linear model with a significant *p*-value (Bonferroni-corrected α = 0.05/29= 1.72.10^-3^), *r*^2^ and *p*-values are shown, classified according to *p*-values.

## Abbreviations

ANOVA: analysis of variance
AUC: area under curve
DAD: diode array analysis
DMSO: dimethylsulfoxide
HMBC: heteronuclear multiple-bond correlation
HMQC: heteronuclear multiple-quantum correlation
HPLC: high precision liquid chromatography HSD: Honest significant difference
NLA: necrotic leaf area
NMR: nuclear magnetic resonance
OE: organic extract
OEW: organic extract weight
QDR: quantitative disease resistance
QRL: quantitative resistance locus
QTL: quantitative trait locus
RE: raw extract
