# Supplementary figures and images for: Aldaulactone – An Original Phytotoxic Secondary Metabolite Involved in the Aggressiveness of Alternaria dauci on Carrot
Source: Front Plant Sci. 2018 May 3;9:502. doi: 10.3389/fpls.2018.00502 (PMC5943595; doi:10.3389/fpls.2018.00502)

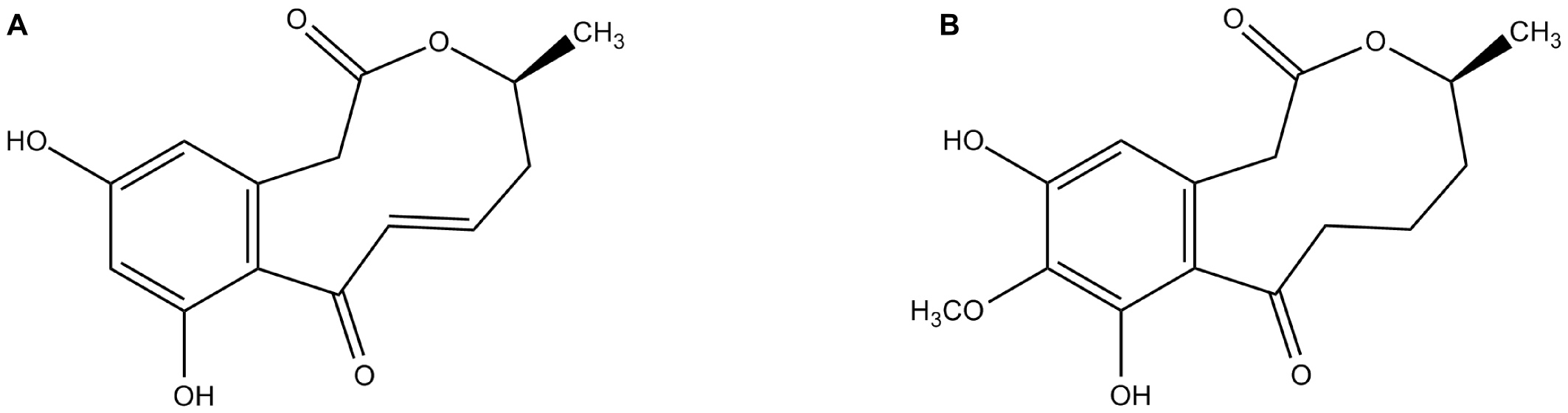

Supplement: FIGURE S1 — Compounds structurally related to aldaulactone. (A) Sporostatin; (B) xestodecalactone D. [file Image_1.JPEG]
