# Supplementary material for: Aldaulactone – An Original Phytotoxic Secondary Metabolite Involved in the Aggressiveness of Alternaria dauci on Carrot
Source: Front Plant Sci. 2018 May 3;9:502. doi: 10.3389/fpls.2018.00502 (PMC5943595; doi:10.3389/fpls.2018.00502)
Supplement: TABLE S1 — List of characterized genes corresponding to quantitative disease resistance loci. This table sums up literature presented previously (Poland et al., 2009; Lecomte et al., 2014; French et al., 2016) or not (for At5g22540, qPLSr5a, and pan1). Mixed-up references presented in (French et al., 2016) have been sorted out. [file Table_1.DOCX]

Supplementary Material

Aldaulactone – an original phytotoxic secondary metabolite involved in the aggressiveness of *Alternaria dauci* on carrot

Julia Courtial, Latifa Hamama, Jean-Jacques Helesbeux, Mickaël Lecomte, Yann Renaux, Esteban Guichard, Linda Voisine, Claire Yovanopoulos, Bruno Hamon, Laurent Ogé, Pascal Richomme, Mathilde Briard, Tristan Boureau, Séverine Gagné, Pascal Poupard and Romain Berruyer*

*** Correspondence:** [romain.berruyer@univ-angers.fr](mailto:romain.berruyer@univ-angers.fr)

**Supplementary Table 1. List of characterized genes corresponding to quantitative disease resistance loci.** This table sums up literature presented previously (French et al., 2016; Lecomte et al., 2014; Poland et al., 2009) or not (for *At5g22540*, *qPLSr5a*, and *pan1*). Mixed-up references presented in (French et al., 2016) have been sorted out.

| gene | type | pathogen | plant | Reference | Category^a^ |
| --- | --- | --- | --- | --- | --- |
| *ZmWAK* | wall-associated kinase, non-RD | *Sporisorium reilianum* | maize | (Zuo et al., 2015) | 2, 5^b^ |
| *Htn1* | wall-associated kinase, non-RD | *Exserohilum turcicum* | maize | (Hurni et al., 2015) | 2, 5 |
| *Yr36* | kinase with START domain | *Puccinia striiformis* | wheat | (Fu et al., 2009) | 6 |
| *RFO1 / WAKL22* | wall associated kinase-like | *Fusarium oxysporum* f. sp. *matthioli* | *Arabidopsis* | (Diener and Ausubel, 2005) | 2, 5 |
| *RKS1* | atypical kinase | *Xanthomonas campestris* pv *campestris* | *Arabidopsis* | (Huard-Chauveau et al., 2013) | 6 |
| *Lr34* | putative ABC transporter | *P. striiformis, Puccinia triticina, Blumeria graminis* | wheat | (Chauhan et al., 2015) | 6 |
| *pi21* | proline containing protein | *Magnaporthe oryzae* | rice | (Fukuoka et al., 2009) | 6 |
| *Rhg4* | serine hydroxy-methyl transferase | *Heterodera glycines* | soybean | (Liu et al., 2012) | 6 |
| *Rhg1* | three ORF, an aa transporter, an alpha SNAP protein and a wound inducible protein | *H. glycines* | soybean | (Cook et al., 2012) | 6 |
| *Pi34* | unknown | *M. oryzae* | rice | (Zenbayashi-Sawata et al., 2007) | 6 |
| *Pi35* | NBS-LRR | *M. oryzae* | rice | (Fukuoka et al., 2014) | 5 |
| *RPS4* | gene pair: NBS-LRR and NBS-LRR-WRKY | *Xanthomonas campestris* pv *campestris* | *Arabidopsis* | (Debieu et al., 2016) | 5 |
| *At5g22540* | unknown | *X.campestris* pv *campestris* | *Arabidopsis* | (Debieu et al., 2016) | 6 |
| *Chr8 QTL* | complex locus, Os-GLP cluster, germin-like proteins | *M.oryzae* | rice | (Manosalva et al., 2009) | 6^c^ |
| *qBlsr5a(1)* | recessive resistance gene xa5 | *Xanthomonas oryzae* pv *oryzicola* | rice | (Xie et al., 2014) | 5 |
| *qBlsr5a(2)* | polygalacturonase-inhibiting protein | *X. oryzae* pv *oryzicola* | rice | (Feng et al., 2016) | 2 |
| *BSR1* | receptor-like cytoplasmic kinase | *Pseudomonas syringae* | *Arabidopsis* | (Dubouzet et al., 2011) | 2, 5 |
| *Pm-21* | serine/threonine kinase | *Blumeria grazminis* f. sp. *tritici* | wheat | (Cao et al., 2011) | 6 |
| *pan1* | receptor-like kinase | *E. turcicum*, *Pantoea stewartii* | maize | (Jamann et al., 2014) | 2, 5 |

^a^Categories are numbered according to the mechanisms proposed in ([Poland et al., 2009](#_ENREF_35)).1: morphological variation, 2: involvement of microbial triggered immunity, 3: involvement of chemical warfare, 4: involvement of signal transduction pathways involved in effector triggered immunity, 5: QRLs as weak version of the resistance genes, and 6: new mechanisms. ^b^*ZmWAK, Htn,1 RFO1 / WAKL22, BSR1and pan1* show homologies with both resistance genes and pathogen reconnaissance protein genes involved in microbial triggered immunity. ^c^GLPs are known plant defense proteins. Although not new, this mechanism was not classified by Poland et al. (2009).
