# Supplementary material for: Aldaulactone – An Original Phytotoxic Secondary Metabolite Involved in the Aggressiveness of Alternaria dauci on Carrot
Source: Front Plant Sci. 2018 May 3;9:502. doi: 10.3389/fpls.2018.00502 (PMC5943595; doi:10.3389/fpls.2018.00502)
Supplement: TABLE S2 — HPLC analysis of fungal organic extracts: correlations with aggressiveness. Organic extract from three different cultures of A. dauci AUS001, FRA001, FRA017, and ITA002 strains and the A. brassicicola Abra 43 strain were analyzed using HPLC-DAD. Seven major peaks were selected for area under a curve (AUC) statistical analysis. Eleven candidate compounds were derived from those peaks, as described in the section “Materials and Methods.” OEW, and AUC and OEW × AUC for each candidate compound were used as variables in 23 linear models of aggressiveness based on data obtained previously [mean NLA in Boedo et al. (2012), Table 3]. For each linear model with a significant p-value (Bonferroni-corrected α = 0.05/29= 1.72.10-3), r2 and p-values are shown, classified according to p-values. [file Table_2.DOCX]

Supplementary Material

Aldaulactone – an original phytotoxic secondary metabolite involved in the aggressiveness of *Alternaria dauci* on carrot

Julia Courtial, Latifa Hamama, Jean-Jacques Helesbeux, Mickaël Lecomte, Yann Renaux, Esteban Guichard, Linda Voisine, Claire Yovanopoulos, Bruno Hamon, Laurent Ogé, Pascal Richomme, Mathilde Briard, Tristan Boureau, Séverine Gagné, Pascal Poupard and Romain Berruyer*

*** Correspondence:** romain.berruyer@univ-angers.fr

**Supplementary Table 2. HPLC analysis of fungal organic extracts: correlations with aggressiveness**. Organic extract from three different cultures of *A. dauci* AUS001, FRA001, FRA017 and ITA002 strains and the *A. brassicicola* Abra 43 strain were analyzed using HPLC-DAD. Seven major peaks were selected for area under a curve (AUC) statistical analysis. Eleven candidate compounds were derived from those peaks, as described in the Material and Methods. OEW, and AUC and OEW×AUC for each candidate compound were used as variables in 23 linear models of aggressiveness based on data obtained previously (mean NLA in ([Boedo et al., 2012](#_ENREF_3)), Table 3). For each linear model with a significant p-value (Bonferoni-corrected α = 0.05/29= 1·72.10^-3^), r² and p-values are shown, classified according to p-values.

| variable | r² | p |
| --- | --- | --- |
| AUC 6m^a^ | 0.9014 | <2.20·10^-16^ |
| OEW×AUC 6m | 0.8337 | 1.30·10^-13^ |
| OEW×AUC 4m | 0.7516 | 6.84·10^-11^ |
| OEW | 0.7442 | 1.09·10^-10^ |
| AUC 5c | 0.6629 | 8.23·10^-9^ |
| OEW×AUC 5c | 0.6614 | 8.82·10^-9^ |
| AUC 4m | 0.6610 | 9.01·10^-9^ |
| OEW×AUC 1a | 0.5678 | 4.16·10^-7^ |
| OEW×AUC 3b | 0.4474 | 2.09·10^-5^ |
| OEW×AUC 2a | 0.3794 | 1.36·10^-4^ |
| AUC 5b | 0.3477 | 3.05·10^-4^ |
| AUC 1c | 0.3375 | 3.93·10^-4^ |
| OEW×AUC 7m | 0.3218 | 5.76·10^-4^ |
| OEW×AUC 5b | 0.3135 | 7.03·10^-4^ |

^a^OEW: Organic extract weight, AUC: area under a curve (in the HPLC profiles), 1-7: peak number as in Table 3, a: 233 nm AUC, b: 254 nm AUC, c: 285 nm AUC, m: (a+b+c)/3.
